# Systemic versus local adipokine expression differs in a combined obesity and osteoarthritis mouse model

**DOI:** 10.1038/s41598-021-96545-8

**Published:** 2021-08-20

**Authors:** Marie-Lisa Hülser, Yubin Luo, Klaus Frommer, Rebecca Hasseli, Kernt Köhler, Magnus Diller, Lina Van Nie, Christoph Rummel, Martin Roderfeld, Elke Roeb, Georg Schett, Aline Bozec, Ulf Müller-Ladner, Elena Neumann

**Affiliations:** 1 Department of Rheumatology and Clinical Immunology, Campus Kerckhoff, Justus-Liebig-University of Giessen, Bad Nauheim, Germany; 2 Department of Medicine 3, Rheumatology and Immunology, Friedrich Alexander University Erlangen-Nuremberg and Universitätsklinikum, Erlangen, Germany; 3 Institute of Veterinary Pathology, Justus-Liebig-University of Giessen, Giessen, Germany; 4 Institute of Veterinary Physiology and Biochemistry, Justus-Liebig-University of Giessen, Giessen, Germany; 5 Department of Gastroenterology, Justus-Liebig-University of Giessen, Giessen, Germany; 6 Department of Rheumatology and Clinical Immunology, Campus Kerckhoff, Justus-Liebig-University of Giessen, Benekestr. 2-8, 61231 Bad Nauheim, Germany

**Keywords:** Immunology, Rheumatology

## Abstract

Osteoarthritis (OA) is a degenerative joint disease characterized by cartilage loss and reduced joint function. OA risk factors are age and obesity. Many adipokines are altered by obesity but also OA although systemic adipokine regulation in OA is not always clear. Therefore, metabolic effects of diet-induced obesity on OA development as well as the influence of obesity and OA progression on systemic vs. local adipokine expression in joints were compared. C57Bl/6-mice fed with HFD (high fat diet) or normal diet prior to destabilization of the medial meniscus (DMM) were sacrificed 4/6/8 weeks after surgery. Sera were evaluated for adiponectin, leptin, visfatin, cytokines. Liver grading and staging for non-alcoholic steatohepatitis (NASH) was performed and crown-like structures (CLS) in adipose tissue measured. OA progression was scored histologically. Adipokine-expressing cells and types were evaluated by immunohistochemistry. Time-dependent changes in DMM-progression were reflected by increased systemic adiponectin levels in DMM especially combined with HFD. While HFD increased serum leptin, DMM reduced systemic leptin significantly. OA scores correlated with bodyweight, leptin and hepatic scoring. Locally, increased numbers of adiponectin- and leptin-producing fibroblasts were observed in damaged menisci but visfatin was not changed. Local adipokine expression was independent from systemic levels, suggesting different mechanisms of action.

## Introduction

Osteoarthritis (OA) is a common degenerative joint disease characterized by loss of cartilage matrix and an irreversible reduction of joint function. The most frequently affected joints are hands, knees, hips and the spinal column^1^. Common OA risk factors are age and long-term mechanical use e.g. caused by sports or obesity, aggravating not only the incidence but also disease progression^2^. Injuries of bone or cartilage also lead to higher risk for OA^3^. Physical wear cannot fully explain the pathogenesis as some joints rarely show signs of OA. Inflammatory processes have been shown to enhance OA cartilage degradation^4^. Loss of cartilage is not the only feature of OA, the surrounding tissues like bone or synovium are affected as well^2^. Local synovitis leads to enhanced proliferation of synovial fibroblasts (SF) and hypertrophic tissue formation with increased angiogenesis as features of advanced OA. However, synovial inflammation in OA may be a secondary effect of continuous tissue degradation^5^. Interestingly, a recent study showed a detrimental effect of parental HFD and obesity on musculoskeletal integrity of two generations of offspring in mice fed with high fat diet (HFD)^6^.

Obesity leads to higher physical load of the joints and influences inflammation by regulating cytokine and adipokine levels^7^. Adipokines including adiponectin, leptin, or visfatin are biologically active factors synthesized mainly by adipocytes^8,9^. Serum adiponectin concentrations in mice and humans are negatively correlated with BMI and bodyweight in healthy individuals^10,11^. In vitro, adiponectin induces cytokine secretion such as interleukin (IL)-6, pro-destructive matrix-metalloproteinase (MMP)-1^12^ and prostaglandin E2 (PGE_2_) in OASF^13^. Systemic leptin levels correlate with bodyweight in mice and humans^14^. Leptin increases IL-6 and IL-8 secretion by OASF via processes involving insulin receptor substrate 1 (IRS-1), phosphatidylinositol-3-kinases (PI3K), protein kinase B (Akt) and activator protein 1 (AP-1) induced for IL-6^15^ or NFκB induced for IL-8^16^. Some studies showed that not only weight bearing joints such as knee and hip are frequently affected in obese patients, but also non-weight-bearing joints such as fingers^2^. Whether this is a consequence of altered adipokine levels is not fully elucidated^2^.

To evaluate adipokines in OA, a mouse model has to provide high reproducibility and similarity to human OA and a slow progression to imitate the human situation. In humans, retrospective studies investigating patients with injured anterior cruciate ligament showed that these patients developed knee OA over the following years^17,18^. This phenomenon could be reproduced in mouse models by surgical dissection of the medial menisci (DMM)^19^. Due to the slow progression, the influence of adipokines can be displayed with higher resolution compared to other OA models. The HFD model is well characterized in C57Bl/6 mice, representing different phenomena observed in humans, e.g. inducing obesity and insulin resistance^20–22^. HFD markedly enhanced osteoarthritis progression and cartilage destruction in the DMM model^23,24^. It could be shown that the dietary fatty acid composition plays an important role^25–27^. We used the combination of both models to evaluate if systemic adipokine changes due to obesity influence local adipokines in the joints or if local adipokines mainly react to local joint degradation over time. These comparisons between systemic and local effects were done at different stages of OA and local cell types affected by the differentially regulated adipokines were characterized.

## Results

### OA course

**Figure 1:**
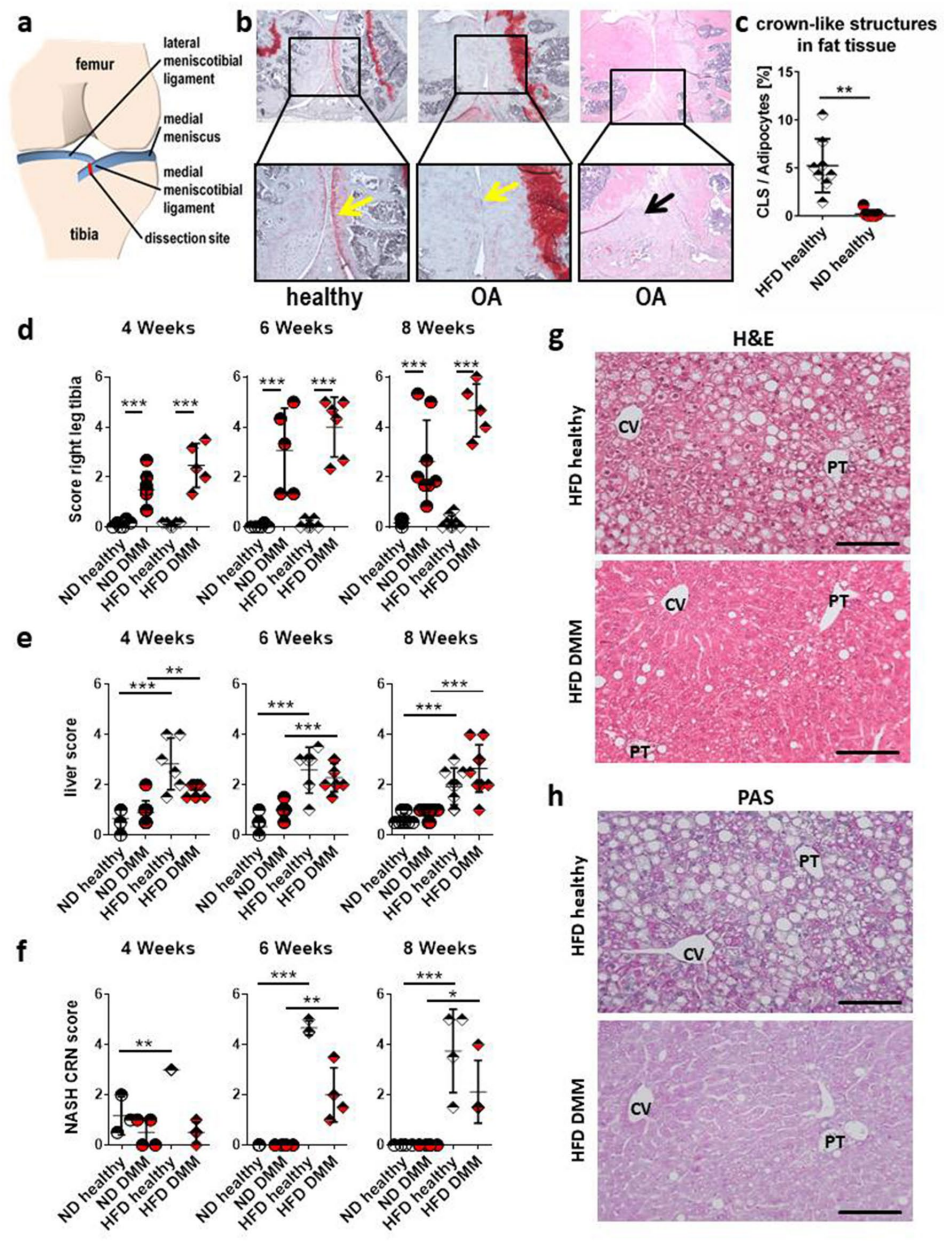
Combination of the DMM and the HFD mouse model. (**a**) Overview and cutting site of the medial meniscus leading to destabilization and arthritis in the DMM model. (**b**) Histological staining (Safranin-O left, middle; H/E right) for histological scoring. Yellow arrows indicate areas of healthy or damaged/missing cartilage; black arrow indicating cell invasion into the damaged meniscus. 50-fold/100-fold magnification. (**c**) Quantified crown-like structures in adipose tissue of HFD vs. ND animals (ND n = 7, HFD n = 8). (**d**) OA scores of the tibia representing arthritis induction and joint degradation at all time points (4 weeks: ND h (healthy) n = 6, ND DMM n = 7, HFD h n = 6, HFD DMM n = 5; 6 weeks: ND h n = 6, ND DMM n = 5, HFD h n = 6, HFD DMM n = 6; 8 weeks: ND h n = 7, ND DMM n = 8, HFD h n = 8, HFD DMM n = 5). (**e**) Fatty liver score confirming metabolic changes due to HFD in both models (4 + 6 weeks: ND h n = 6, ND DMM n = 10, HFD h n = 6, HFD DMM 4 weeks: n = 10; 6 weeks: n = 7. 8 weeks: ND h n = 8, ND DMM n = 10, HFD h n = 8, HFD DMM n = 10). (**f**) Quantification of the NASH-CRN-score shows an increased score under HFD at weeks 6 and 8 which is reduced by DMM at all time points (n = 4, except n = 3 for ND healthy, HFD healthy at 4 and 6 weeks, respectively). (**g**) Representative H/E staining of the liver showing increased hepatic fat in HFD animals (upper panel) while hepatic fat was reduced in animals fed with HFD and DMM (lower panel) at early time points. (**h**) Mice fed with HFD showed increased glycogen accumulation in the liver (upper panel) which was reduced in animals fed with HFD and DMM (lower panel). CV: central vein, PT: portal tract. ×200 magnification, bar: 100 µm. Statistic was performed with unpaired parametric t-test with Welch´s correction (**c**) and two-way ANOVA followed by Sidak’s multiple comparison test for post-hoc analysis (**d**–**f**). The number of animals involved in each experiment is summarized in Table 2.

Animals were fed with HFD or matched normal diet (ND) for 3 months prior to surgical OA induction by DMM (Fig. 1a). Controls (healthy, untreated animals) and non-surgical sham-treated control limbs from DMM animals are shown in supplement 1. Tibia as well as femur of all animals was scored. Due to similar values (supplement 1), tibia scores are shown and used for correlation analysis. Histological joints analysis (Fig. 1b) showed a significant OA induction at all time points comparing DMM with healthy controls (Fig. 1d). At week 4, ND animals with DMM showed OA scores of 1.50 ± 0.7 (n = 7) compared to healthy ND animals with 0.11 ± 0.14 (n = 6; p = 0.0005). HFD animals with DMM scored with 2.47 ± 0.88 (n = 5) compared to healthy HFD-fed animals (0.11 ± 0.09; n = 6, p < 0.0001) 4 weeks after induction. 6 weeks after surgery, the ND group showed an OA score of 3.06 ± 1.69 (n = 5) vs. ND healthy of 0.03 ± 0.07 (n = 6, p = 0.0001), whereas the HFD group had a higher score of 4 ± 1.19 (n = 6) compared to HFD healthy (0.11 ± 0.17; n = 6; p < 0.0001). OA progression between 4 and 6 weeks was stronger than 6 to 8 weeks. 8 weeks after surgery the OA score in ND mice was 2.63 ± 1.65 (n = 8) compared to ND healthy controls 0.17 ± 0.13 (n = 7, p = 0.0002) and in HFD mice 4.67 ± 1.06 (n = 5) vs. HFD healthy animals 0.19 ± 0.26 (n = 8, p < 0.0001) (Fig. 1d). Statistical significance values for overall effects of each individual factor, i.e. diet and OA induction, as well as the interaction of these factors, as obtained from two-way ANOVA, are shown in supplement 4 (Table 1).

**Table 1:** Systemic adipokine levels in all treatment groups shown for all time points.

Adipokine	Week	ND healthy	ND DMM	HFD healthy	HFD DMM
Adiponectin	4	6191 (± 915.8)	5486 (± 858.2 )	5928 (± 496.1)	5605 (± 246.6)
	6	6625 (± 182.7)	5770 (± 692.9)	5742 (± 943.2)	4952 (± 930.8)
	8	6094 (± 287.7)	6359 (± 273.7)	5176 (± 417.7)	6290 (± 393)
Leptin	4	18.42 (± 9.267)	2.882 (± 1.134)	86.94 (± 16.97)	33.56 (± 17.34)
	6	19.16 (± 3.797)	10.06 (± 7.024)	94.47 (± 10.52)	53.14 (± 32.58)
	8	15.27 (± 5.818)	14.42 (± 8.017)	59.35 (± 31.17)	32.23 (± 10.85)
Visfatin	4	55.52 (± 33.87)	75.86 (± 48.32)	37.56 (± 14.44)	44.47 (± 20.32)
	6	39.17 (± 21.79)	45.64 (± 18.51)	56.53 (± 63.21)	67.26 (± 46.8)
	8	11.34 (± 6.854)	23.41 (± 13.95)	52.32 (± 49.41)	26.19 (± 0.356)

Values are given as mean (± standard deviation) in [ng/ml].

### Histological examinations

Safranin-O staining showed severe cartilage loss with deep defects down to the bone. Cartilage matrix and the dissected menisci were deeply invaded by non-chondrocytic cells, specifically at later time points (Fig. 1b). HFD-induced inflammation in fat tissue was reflected by significantly increased numbers of CLS in HFD mice (5.22 ± 0.98; n = 8) compared to ND (0.2 ± 0.15; n = 7, p = 0.0013; Fig. 1c). HFD induced significant changes in the fatty liver score after 4, 6, and 8 weeks (e.g. ND healthy vs. HFD healthy: 0.63 vs. 1.94, p = 0.0004; ND DMM vs. HFD DMM: 0.9 vs. 2.65, p < 0.0001; Fig. 1e,g). These scores confirmed the clinical effects of the combination of DMM and HFD in an in vivo experimental setting. Pathologic NASH-CRN-scoring revealed a significant increase of the score in healthy HFD animals after 6 and 8 weeks whereas DMM combined with HFD reduced the score at all time points (Fig. 1f,g). PAS staining revealed an increased glycogen accumulation in HFD animals compared to ND (Fig. 1h) and lower glycogen accumulation in HFD animals with DMM. No differences regarding glycogen accumulation were observed between ND control and ND DMM animals. There was no significant change in immune cell infiltration in healthy and DMM animals under ND or HFD.

### Systemic adipokine levels

**Figure 2:**
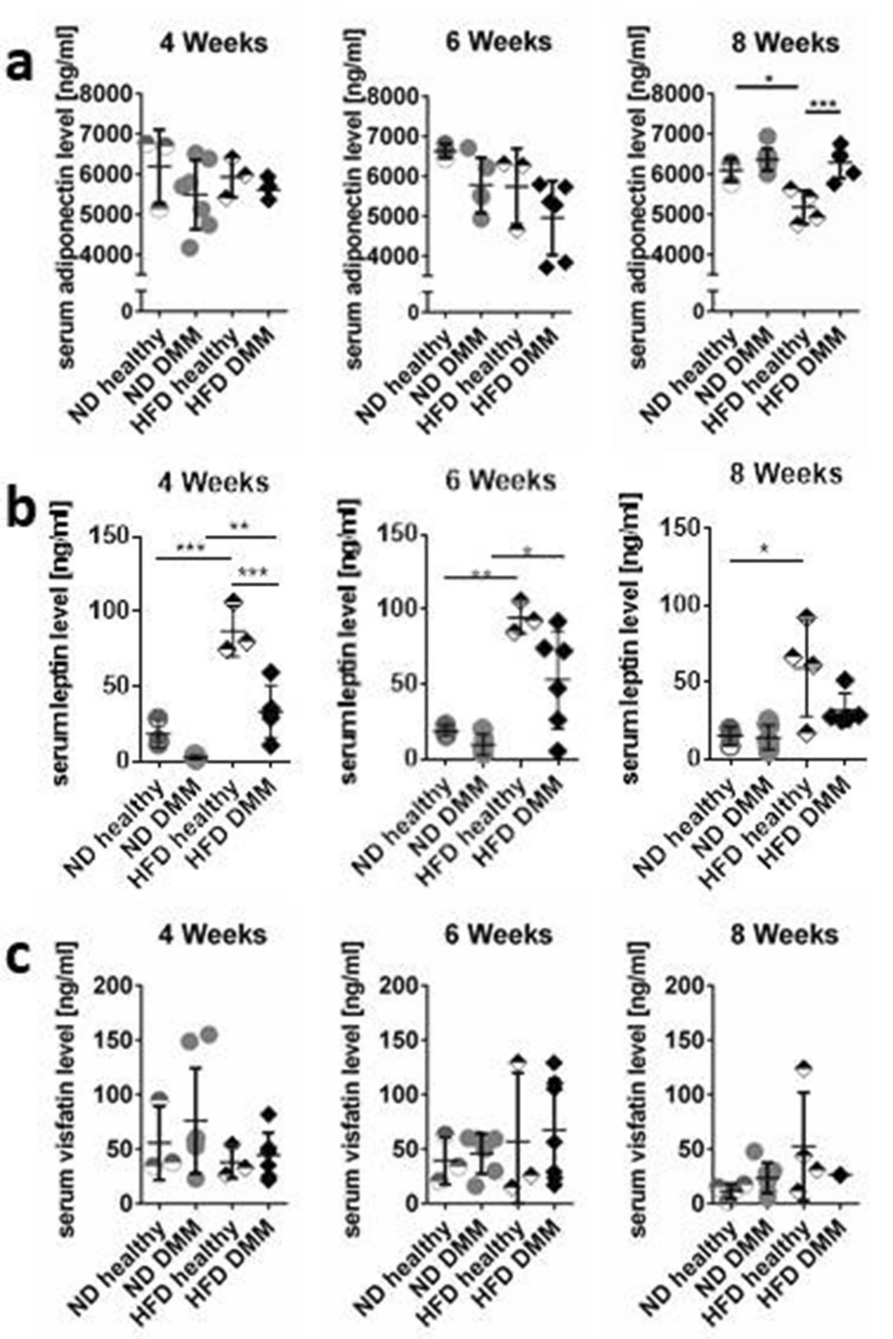
Systemic adipokine levels with or without DMM and HFD. (**a**) Adiponectin was downregulated by HFD with significant reduction in the healthy (h) groups 8 weeks after surgery. DMM-induced adiponectin was visible 8 weeks after surgery (4 weeks: ND h (healthy) n = 3, ND DMM n = 7, HFD h n = 3, HFD DMM n = 5; 6 weeks: ND h n = 3, ND DMM n = 5, HFD h n = 3, HFD DMM n = 6; 8 weeks: ND h n = 3, ND DMM n = 8, HFD h n = 4, HFD DMM n = 5). (**b**) Leptin was significantly induced by HFD compared to ND at all time points. DMM led to a reduction of leptin levels (n-numbers see **a**). (**c**) Visfatin levels were not changed by diet or DMM induction (4 weeks: ND h n = 3, ND DMM n = 8, HFD h n = 3, HFD DMM n = 7; 6 weeks: ND h n = 3, ND DMM n = 6, HFD h n = 3, HFD DMM n = 7; 8 weeks: ND h n = 4, ND DMM n = 7, HFD h n = 4, HFD DMM n = 3). Statistical analysis was performed by two-way ANOVA followed by Sidak’s multiple comparison test for post-hoc analysis.

HFD-induced obesity did not alter systemic adiponectin levels at early time points. Eight weeks after OA-induction a significant adiponectin reduction was detected in HFD-fed healthy animals compared to ND (5176 ± 417.7 ng/ml vs. 6094 ± 287.7 ng/ml; p = 0.0157; Fig. 2a). OA did not change adiponectin at early time points but led to an induction after 8 weeks, which was significantly higher in HFD (HFD DMM: 6290 ± 393 ng/ml vs. HFD healthy 5176 ± 417.7 ng/ml; p = 0.0009; Fig. 2a). Systemic leptin levels were significantly elevated in HFD after 4 and 6 weeks (e.g. 4 weeks ND healthy 18.42 ng/ml vs. HFD healthy 86.94 ng/ml, p =  < 0.0001, ND DMM 2.88 ng/ml vs. HFD DMM 33.56 ng/ml, p = 0.0044) and elevated after 8 weeks but reduced by OA induction. The reduction through OA was visible in every group but only after 4 weeks with HFD statistically significant (Fig. 2b). Interestingly, the variation in individual levels visualized by the standard deviation (SD) was higher in HFD compared to the corresponding ND groups (Table 1). Visfatin levels neither changed over time nor by OA induction or HFD (Fig. 2c). Statistical significance values for overall effects of each individual factor, i.e. diet and OA induction, as well as the interaction of these factors are shown in supplement 4 (Table 2).

**Table 2:** Number of animals involved in each experiment for all groups:

	Week	ND healthy	ND DMM	HFD healthy	HFD DMM
**Figure ****1**					
c) CLS	-	7	–	8	–
d) Scores right leg, tibia	4	6	7	6	5
	6	6	5	6	6
	8	7	8	8	5
e) Liver score	4	6	10	6	10
	6	6	10	6	7
	8	8	10	8	10
f) NASH CRN score	4	3	4	3	4
	6	3	4	3	4
	8	4	4	4	4
**Figure ****2**					
a) Adiponectin & b) Leptin	4	3	7	3	5
	6	3	5	3	6
	8	3	8	4	5
c) Visfatin	4	3	8	3	7
	6	3	6	3	7
	8	4	7	4	3
**Figure ****3**					
a) All animals	4	3	8	3	7
	6	3	6	3	7
	8	4	8	4	5
b) DMM animals	4	n.a.	7	n.a.	5
	6	n.a.	5	n.a.	6
	8	n.a.	8	n.a.	5
c) Bodyweight	4	6	10	6	10
	6	6	10	6	7
	8	8	10	8	10
**Figure ****4**					
a) Adiponectin, leptin & visfatin joint staining	4	3	10	3	10
	6	3	10	3	7
	8	4	10	4	10
b) Adiponectin quantification	4	4	6	5	5
	6	5	7	5	2
	8	1	8	4	10
b) Leptin quantification	4	4	3	5	3
	6	5	5	4	3
	8	2	7	7	9
**Figure ****5**					
F4/80 staining	4	3	10	3	10
	6	3	10	3	7
	8	4	10	4	10

Mean values of experimental replicates were calculated and statistics performed using mean values of each biological replicate (animal). N.a. = not applicable.

### Correlations between metabolic parameters and OA induction

**Figure 3:**
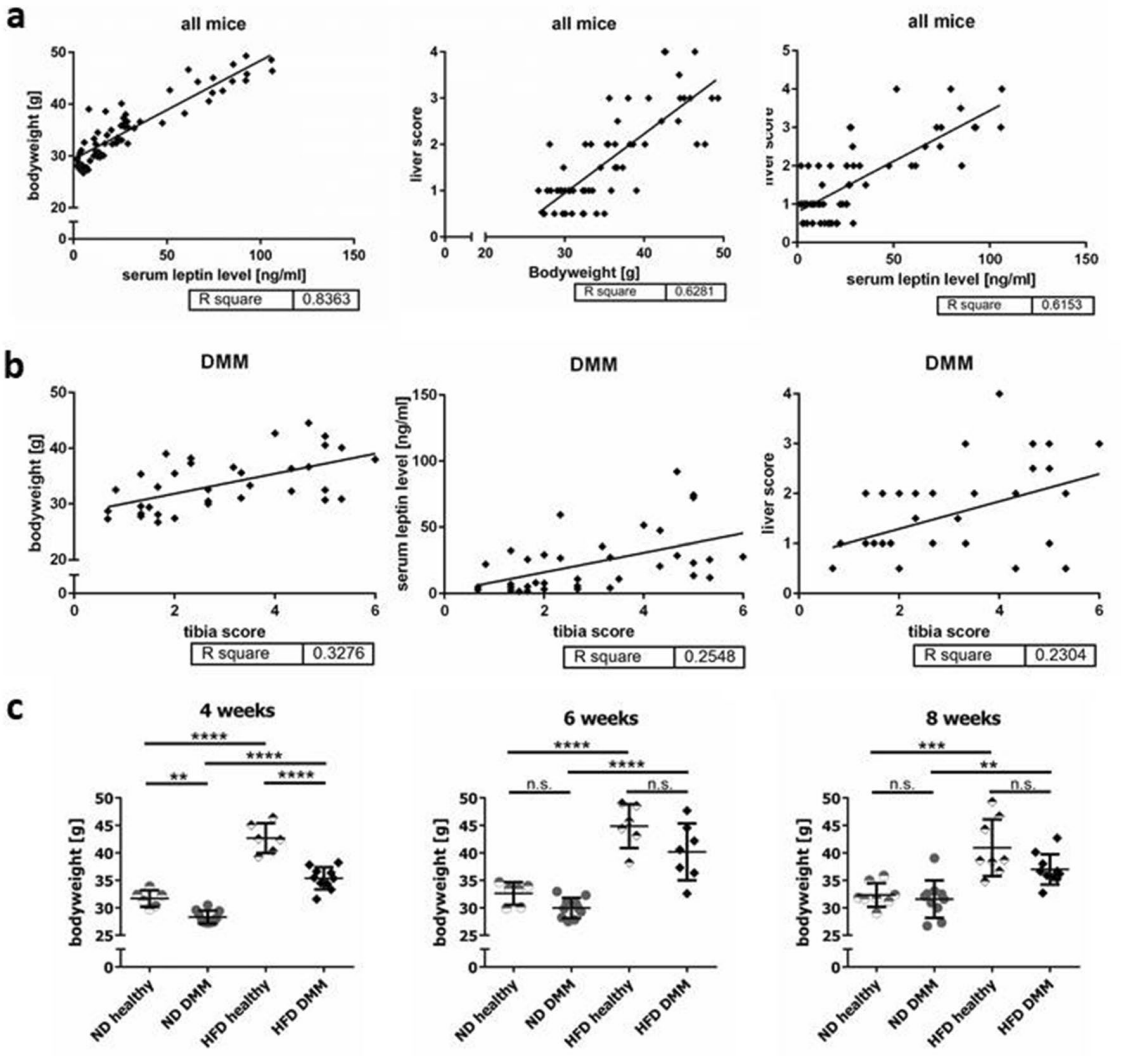
Correlation analysis of metabolic changes with the arthritis score. (**a**) Comparison of all animals (n = 61) regarding metabolic parameters including body weight, liver score, and serum leptin levels revealed correlation of these parameters with each other (4 weeks: ND h (healthy) n = 3, ND DMM n = 8, HFD h n = 3, HFD DMM n = 7; 6 weeks: ND h n = 3, ND DMM n = 6, HFD h n = 3, HFD DMM n = 7; 8 weeks: ND h n = 4, ND DMM n = 8, HFD h n = 4, HFD DMM n = 5). (**b**) In the DMM group (n = 36), the bodyweight correlated best with OA progression, whereas leptin levels and liver score show lower r^2^-values. r^2^ of > 0.3 is considered as significant (4 weeks: ND DMM n = 7, HFD DMM n = 5; 6 weeks: ND DMM n = 5, HFD DMM n = 6; 8 weeks: ND DMM n = 8, HFD DMM n = 5). (**c**) Influence of ND, HFD and DMM induction on bodyweight after 4, 6 and 8 weeks is shown (4 weeks: ND h n = 6, ND DMM n = 10, HFD h n = 6, HFD DMM n = 10; 6 weeks: ND h n = 6, ND DMM n = 10, HFD h n = 6, HFD DMM n = 7; 8 weeks: ND h n = 8, ND DMM n = 10, HFD h n = 8, HFD DMM n = 10).

With respect to all animals, linear regression analysis showed that bodyweight correlated with leptin levels (r^2^ = 0.8363) and to a lower extend with liver scores (r^2^ = 0.6281) (Fig. 3a). Liver score and leptin levels also correlated (r^2^ = 0.6153). Regarding the tibia score, only animals which underwent surgery were evaluated (Fig. 3b). A positive correlation between tibia scores and bodyweight (r^2^ = 0.3276) was observed, and a weaker correlation between tibia scores and systemic leptin (r^2^ = 0.2548) or liver score (r^2^ = 0.2304). Bodyweight of the HFD groups were significantly higher compared to the ND groups at all time points (e.g.: 4 weeks ND healthy vs. HFD healthy 31.68 g ± 1.51 g vs 42.67 g ± 2.74 g, p < 0.0001, Fig. 3c). DMM induction led to less weight gain compared to healthy controls in the ND as well as the HFD group (Fig. 3c).

### Local adipokine synthesis

**Figure 4:**
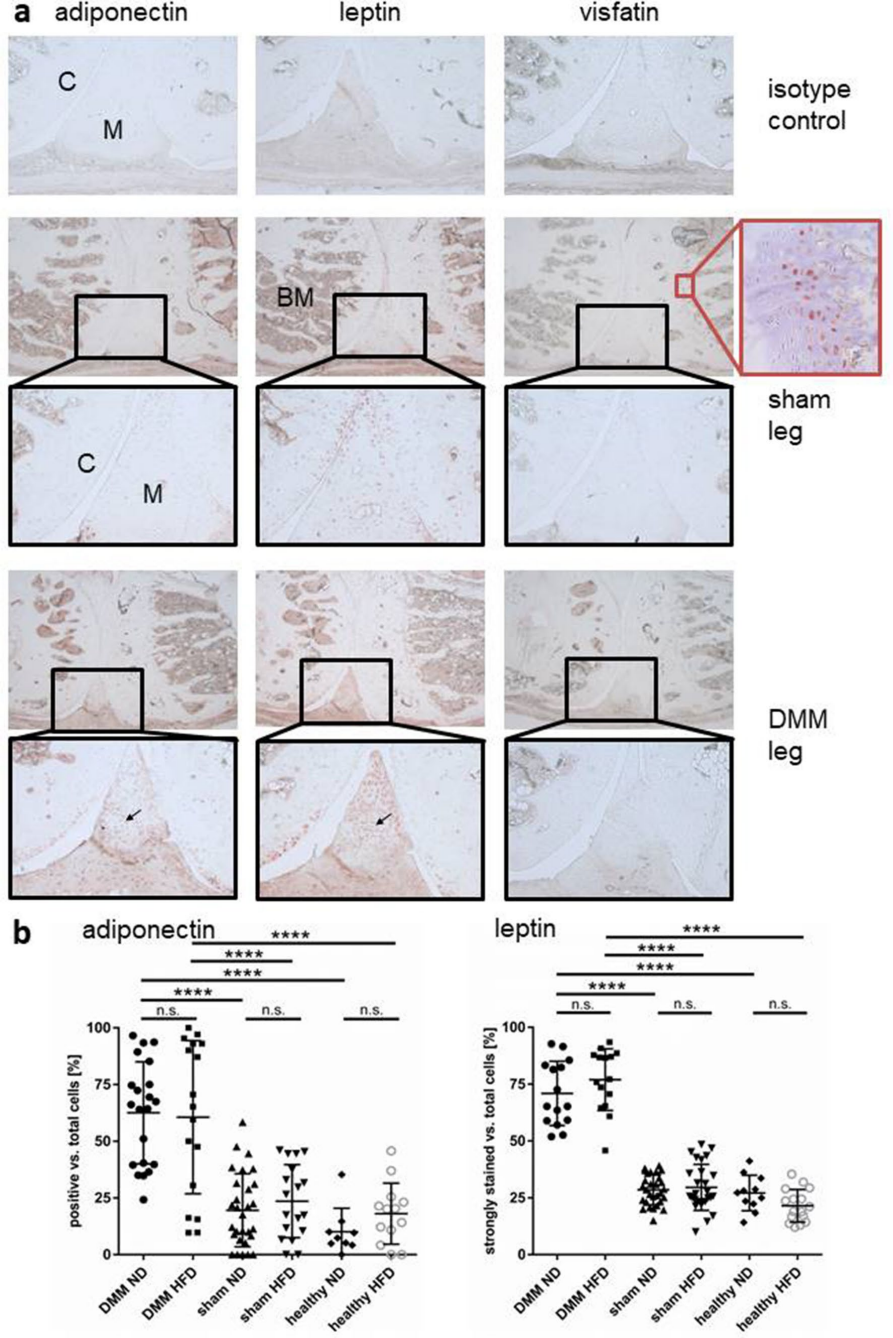
Local adipokine distribution in DMM vs. sham-treated legs. (**a**) All adipokines were present in the bone marrow in sham-treated and DMM legs. Cells invading the damaged menisci in DMM expressed adiponectin as well as leptin (arrows) independent on the diet. Hypertrophic but not proliferative chondrocytes in the epiphyseal growth plate expressed visfatin (4 weeks: ND h (healthy) n = 3, ND DMM n = 10, HFD h n = 3, HFD DMM n = 10; 6 weeks: ND h n = 3, ND DMM n = 10, HFD h n = 3, HFD DMM n = 7; 8 weeks: ND h n = 4, ND DMM n = 10, HFD h n = 4, HFD DMM n = 10). (**b**) Quantification of adipokine-positive cells in the meniscus revealed an increased number of adipokine positive cells in the DMM groups compared to the healthy groups but no differences between HFD vs. ND in the DMM group could be observed. Ages 4, 6, 8 weeks were pooled for each group (adiponectin 4 weeks: ND h n = 4, ND DMM n = 6, HFD h n = 5, HFD DMM n = 5; 6 weeks: ND h n = 5, ND DMM n = 7, HFD h n = 5, HFD DMM n = 2; 8 weeks: ND h n = 1, ND DMM n = 8, HFD h n = 4, HFD DMM n = 10; sham adiponectin ND n = 29, HFD n = 18. Leptin 4 weeks: ND h n = 4, ND DMM n = 3, HFD h n = 5, HFD DMM n = 3; 6 weeks: ND h n = 5, ND DMM n = 5, HFD h n = 4, HFD DMM n = 3; 8 weeks: ND h n = 2, ND DMM n = 7, HFD h n = 7, HFD DMM n = 9, sham leptin ND n = 29, HFD n = 27). C = cartilage, M = meniscus, BM = bone marrow, h = healthy. 50-fold/100-fold magnification.

The local distribution of adipokines (Fig. 4a) in the mouse joints did not differ between the individual time points or between HFD vs. ND. IHC for adiponectin or leptin showed cellular signals within the cartilage of the joint surface, in cells of the bone marrow, and in synovial cells. When comparing healthy and OA menisci, there was a strong cellular invasion into OA menisci. These cells were adiponectin and leptin positive, reflecting the higher number of cells positive for these adipokines in the areas of cartilage loss in DMM (Fig. 4a, arrows).

Visfatin staining showed strong signals in the hypertrophic area of the cartilage with decreasing signals towards the proliferative zone within the epiphyseal growth plate. These findings were visible in all tissues and independent of the tibia score. To show that visfatin was strongly expressed in hypertrophic cells, the same areas were stained for collagen type X, confirming the presence of hypertrophic chondrocytes (Fig. 4a, inset). Adipokine-positive cells vs. the total number of cells within the meniscus were evaluated (supplement 2). An increase of adiponectin positive cells invading the meniscus in the DMM groups compared to controls could be observed. The numbers of leptin-positive cells with a strong signal in the meniscus were increased in the DMM groups compared to the controls. However, no quantitative difference between HFD and ND in the DMM group could be observed for both adipokines (Fig. 4b).

### Characterization of the meniscus-invading cells

**Figure 5:**
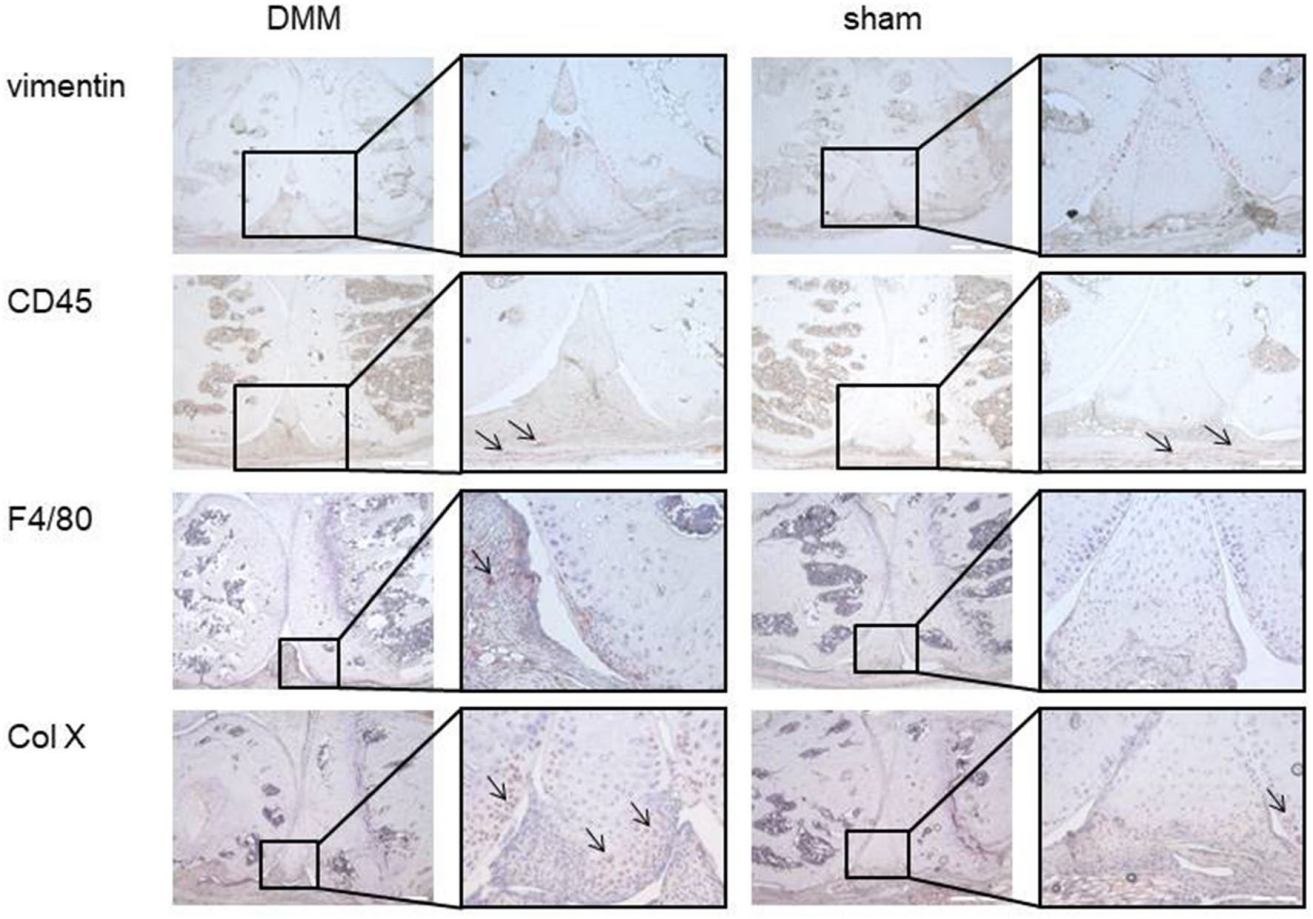
Characterization of cells invading damaged menisci. All cells infiltrating into the meniscus of DMM animals were vimentin positive. IHC with CD45 (leukocyte marker), F4/80 (macrophage marker) or collagen type X (marker for chondrocytes) showed only few positive cells (black arrows) mainly outside of the meniscus indicating most of the cells infiltrating the meniscus are fibroblasts. These results were comparable in DMM or sham surgery (4 weeks: ND h (healthy) n = 3, ND DMM n = 10, HFD h n = 3, HFD DMM n = 10; 6 weeks: ND h n = 3, ND DMM n = 10, HFD h n = 3, HFD DMM n = 7; 8 weeks: ND h n = 4, ND DMM n = 10, HFD h n = 4, HFD DMM n = 10).

Within the menisci of DMM legs, few single cells were positive for the macrophage marker F4/80 in all treatment groups. All invading cells in the menisci showed strong vimentin signals (Fig. 5). This was seen in sham-treated legs as well as in DMM-legs without difference between ND and HFD as shown in representative stainings for each treatment group (n = 4 DMM ND, n = 5 DMM HFD). The leukocyte marker CD45 was expressed only by few solitary cells in the synovium. In total, most of the cells were vimentin positive. Within these cells, we observed two types of cells with morphologically different shape: a smaller number of bigger and round shaped cells and the majority of the cells being small with fibroblast-like shape. The round-shaped and bigger cells were collagen type X positive, identifying them as hypertrophic chondrocytes as shown in representative stainings comparing sham-treated with DMM-treated legs (n = 5 each) (Fig. 5).

### Meniscal ossicle

In some animals, we observed bone-like areas within the menisci. All histological staining characteristics indicate an increase in bone matrix. This phenomenon could be observed randomly in all treatment groups, mainly in DMM legs, but in some cases also in sham legs (Supplement 3). No relation of the observed ossifications to other parameters was detectable.

## Discussion

With respect to the establishment of the experimental setting, the increased presence of lipid droplets in liver cells, the increased NASH-CNS score, and the higher tibia score representing the induced OA showed that both models as well as the combination worked properly. The tibia score was significantly higher in every group compared to the respective healthy control but also higher in HFD vs. ND. Comparing the course of OA progression, the progression in ND mice reached a plateau phase, shown by the small difference in the tibia score between 6 and 8 weeks after surgery. In HFD animals, this plateau was not visible within the evaluated time period, suggesting a higher tibia score for the plateau due to obesity. The increase of CLS in the fat tissue as well as NASH-CRN-score of HFD animals showing the previously described phenomenon of chronic low-grade inflammation^28^ could be confirmed in our models. Xu et al*.*^29^ showed that CLS are formed by macrophages, supporting the idea of a low grade inflammation. Another study comparing HFD to low fat diet in the DMM model showed the highest amounts of CLS with increased M1 macrophages in the DMM HFD group^30^. Fibrosis was also enhanced by DMM with HFD compared to HFD without DMM in this study. In addition, it could be shown that macrophage depletion had beneficial effects in the DMM/HFD model leading to reduced synovitis and cartilage destruction and that intraarticular treatment with resolvin D1 decreased synovial macrophage infiltration, especially of inflammatory macrophages^31^.

The serum leptin induction by HFD observed in our study corresponds to the previously described correlation between leptin and BMI and/or amount of adipose tissue^32^. Serologic studies in humans found a correlation between leptin levels and insulin resistance^33^. Murine studies showed an induction of insulin resistance in C57Bl/6 mice by a comparable HFD^20^. In contrast, healthy ND animals displayed constant leptin levels over time. Animals under ND with DMM showed lower leptin levels at early time points with increasing levels reaching healthy ND values at the latest time point. This indicates that the serum levels were influenced by local processes in the joint showing increasing leptin signals in DMM synovium which leveled out over time. In comparison to ND, HFD showed a higher standard deviation (SD) of individual leptin levels potentially masking the leptin increase observed in ND (Table 1). In human studies, not only an increase in leptin levels by obesity but also an increased SD was described^34^. Therefore, with respect to our experiments with ND or HFD, leptin may not represent a reliable predictive factor or biomarker. However, leptin level under HFD were reduced in animals with DMM compared to healthy HFD animals, which corresponds to the decreased NASH-CRN-score at all time points and liver scores at weeks 4 and 6 after DMM induction.

In contrast to leptin, visfatin levels were homogeneous and unchanged within the groups. The finding that HFD did not alter visfatin was previously described in a comparable mouse model^35^. Additionally, serum visfatin levels positively correlated with non-alcoholic fatty liver progression^36^. However, this could not be confirmed in our mouse model. The tibia score, representing arthritis induction within the treated joint, correlated mainly with bodyweight but not with visfatin. This may be in line with results of human studies showing the strongest effects of obesity in biomechanical wear in the weight bearing knee and hip joints^7^.

Interestingly, systemic adiponectin levels were influenced mainly by OA at the latest time point and only significant in combination with HFD, suggesting that the adiponectin induction is dependent on the stage of OA progression. Systemic inflammatory markers were not increased in this model as shown for IL-6, excluding that increased circulating adiponectin-levels in our model were due to strong systemic inflammation. Since adiponectin exists in many isoforms^14^ it would be interesting for future studies to characterize the composition of adiponectin isoforms induced in late-stage OA.

To compare the systemic adipokine level with the local expression in arthritic joints, IHC was performed showing that all adipokines were present in areas in close exchange with the blood as e.g. the bone marrow in all treatment groups. Our data show that within the damaged menisci, the number of adiponectin and leptin producing cells was increased, resulting in higher expression of adipokines locally at sites of matrix loss. With regard to the systemically strong upregulation of leptin levels due to HFD and downregulation due to DMM, which were not visible locally, it seems that local destructive processes within the joint impact local adipokine levels. This observation matches with human studies describing a positive correlation of leptin levels in the synovial fluid with radiographic OA progression^37^.

In our study, visfatin was specifically observed within the epiphyseal growth plate of the tibia, showing decreasing signals from the hypertrophic to the proliferative zone, which was independent from diet or joint destruction. This suggests a general characteristic previously described within human osteophytes^38^. Other groups suggested a local role of visfatin, which could depend on the individual joint. For example, in human hip OA, a positive correlation between visfatin and pain levels of the patients was described, whereas in knee OA only leptin correlated with pain levels^39^.

The majority of adipokine-expressing cells invading into the damaged menisci in our model were identified as SF showing a strong signal for vimentin and exclusion of other mesenchymal and inflammatory cells. These SF may be responsible for the local expression of adipokines within the joint. Due to the close localization of SF to each other, autocrine as well as paracrine influences of adipokines on SF could be responsible for the alterations observed within the menisci. As previously described, the local effects are mainly pro-inflammatory although adiponectin has anti-inflammatory effects in the periphery, e.g. in cardiovascular diseases^40^. Adiponectin leads to expression of ICAM-1, improves monocyte adherence in the OA synovium and increases secretion of IL-6, proMMP-1 and PGE_2_ in OASF in vitro^12,13,41^. Leptin or visfatin stimulation of OASF increased the secretion of proinflammatory cytokines like IL-6, IL-8, and TNF-α^15,16,42^. IL-8 and MCP-1 were also upregulated by visfatin in a fibroblast cell line^43^. Systemic adipokine level seem to be mainly responsible for energy homeostasis as mice lacking the leptin receptor display massive obesity^44^ and on the other hand adiponectin knockout mice seem to be more susceptible to diet-induced insulin resistance^45^. However, miR-34a-5p was recently shown to be upregulated in plasma and knee joints of DMM mice fed with HFD^46^. This and other studies suggest that epigenetic changes may contribute to lasting pro-osteoarthritic effects induced by HFD. It was recently shown in the DMM model that mice first fed with HFD followed by a switch to ND reduced body weight, restored metabolic parameters and led to less systemic and synovial tissue inflammation^47^ showing the role of inflammatory factors induced for example by adipokines.

Interestingly, ossification of menisci was observed in some animals, which was comparably described in case reports in patients^48–51^. The molecular mechanisms involved in ossicle formation are not well known. In rodents these ossicles seem to be more common compared to humans e.g. as described in a guinea pig OA model^52^. Additionally, they are in part visible in histological figures of murine OA models even though not explicitly mentioned^19^. In a DMM model in rats, HFD increased arthritis scores for all knee surfaces^53^. In this study, the lateral meniscus showed more bone than the medial meniscus but there was no relationship between diets or surgery groups in this study similar to our observations in the DMM treated animals.

In conclusion, both murine models not only reflect the clinical course of OA and obesity in humans, they also show that OA is deteriorated by HFD correlating mainly with bodyweight and to a lower extend with metabolic changes induced by obesity. Interestingly, local adipokine expression was independent from systemic adipokine levels, suggesting different mechanisms of action locally in joints. Nevertheless, obesity as a systemic phenomenon needs further evaluation especially with regard to rheumatic diseases as there are connections between obesity, inflammation, disease progression, and risk^2,54^.

### Limitations

Following the RRR concept, the number of animals was calculated as low as possible leaving the experiments to the absolute minimum necessary for the key questions, which includes also the problem of some animals without successful dissection of the medial meniscus. Furthermore, only male mice were used for our study which also represents a limitation of our study. Systemic adipokine levels were not correlated to the amount of body fat of the animals, which was not quantified in our study, but with the weight of the animals and metabolic parameters such as CLS and NASH-score.

## Materials and methods

### Animal model

10 week old male C57BL/6JRjErl mice were housed under standardized conditions following the guidelines of the German Animal Welfare Act and kept on a normal diet (ND) (D12329, Research Diets) or HFD (D12331, Research Diets) with 58% fat (mostly saturated fatty acids) and water ad libitum for 3 month before DMM surgery on a 12-h light/dark routine. OA-induction was performed by DMM surgery, which destabilizes the knee joint by transsection of the medial meniscus^19^. OA severity was scored as described by Glasson et al*.*^55^. Animal experiments were performed in accordance with the German Animal Welfare Act and international legislation (Directive 2010/63, European Community) and approved by the local government authorities, RP Mittelfranken, Germany, protocol number V54-2532.1-44/12. The experiments were performed in accordance with the ARRIVE guidelines. The numbers of animals involved in the respective experiments are indicated in the legends and summarized in Table 2.

### Histology and histological quantification

Tissues were fixed in 4% formalin (Carl Roth, Karlsruhe, Germany) for 24 h and joints decalcified in NaEDTA (Carl Roth) for 6 weeks. Tissues were dehydrated using ascending ethanol/PBS solutions and xylene, and paraffin embedding. Due to the decalcification period, proteoglycan loss was not quantified due to leaching artefacts. Cartilage loss as well as cartilage and bone erosions were evaluated. Several sections per tissue were evaluated. However, only one value was calculated and used for statistical evaluation of the score (biological replicates). 5 µm sections were stained with hematoxylin and eosin (H/E) (joints/liver), safranin-O (joints), toluidine blue (fat), or periodic acid-Schiff (PAS) staining for glycogen detection (liver). Fatty liver score quantifying the ratio between hepatocytes with and without fat vacuoles was performed (score 0–4)^56^. The NASH-CRN-score (0–12) was utilized for histopathologic grading and staging of nonalcoholic fatty liver disease^57,58^ utilizing the NASH-CRN scoring system comprising 14 histological features, 4 of which were evaluated semi-quantitatively: steatosis (0–3), lobular inflammation (0–3), hepatocellular ballooning (0–2), and fibrosis (0–4). Another nine features were recorded as present or absent. Crown-like structures (CLS) in adipose tissue were quantified counting the adipocytes and infiltrated immune cells per visual field using the ImageJ software and calculating the percentage of CLS per number of adipocytes^29^.

### Immunohistochemistry (IHC)

5 µm joints sections were deparaffinized and antigens retrieved by heat (10 mM sodium citrate, 0.05% Tween20, pH6; 60 min 65 °C) or proteinase K (5 min 37 °C). Endogenous peroxidases were blocked with 3% H_2_O_2_ in methanol, unspecific bindings with 5% BSA. Primary antibodies: leptin (ab3583, abcam, Cambridge, UK), visfatin/PBEF (H-300, sc-67020, Santa Cruz, Dallas, USA), adiponectin/Acrp30 (AF1119, R&D Systems, Minneapolis, USA), F4/80 (abdserotec, Hercules, USA), CD45 (30-F11, Novusbio, Littleton, USA), collagen type X (Abbiotec, San Diego, USA), vimentin (R&D). Secondary antibody system: Histofine^®^ (Nichirei Biosciences, Tokyo, Japan). Isotype and negative controls were performed.

### Immunoassays

Serum adipokine levels were measured using mouse adiponectin/Acrp30, mouse IL-6, mouse/rat leptin Quantikine ELISA (R&D) and visfatin (NAMPT) mouse/rat ELISA (BioVendor, Brno, Czech Republic).

### Statistics

Unpaired parametric t-test with Welch´s correction was used for the calculation of the analysis of the data for the crown-like (CLS) structures, comparing the effect of a ND and HFD in non-arthritic (healthy) mice. Data shown in Figs.1d,e,h, 2 were evaluated using two-way ANOVA followed by Sidak’s test multiple comparison test for post-hoc analysis. All values represent mean ± standard deviation if not declared differently. Significance: p < 0.05 = *; p < 0.01 = **; p < 0.001 = ***; Linear regression analysis: r^2^ > 0.3.

## Supplementary Information

Supplementary Information 1.

Supplementary Information 2.

Supplementary Information 3.

Supplementary Information 4.

## References

[1] Goldring MB, Goldring SR (2007). Osteoarthritis. J. Cell Physiol..

[2] Glyn-Jones S (2015). Osteoarthritis. Lancet.

[3] Muthuri SG, McWilliams DF, Doherty M, Zhang W (2011). History of knee injuries and knee osteoarthritis: A meta-analysis of observational studies. Osteoarthr. Cartil..

[4] Loeser RF, Goldring SR, Scanzello CR, Goldring MB (2012). Osteoarthritis: A disease of the joint as an organ. Arthritis Rheum..

[5] Scanzello CR, Goldring SR (2012). The role of synovitis in osteoarthritis pathogenesis. Bone.

[6] Harasymowicz NS (2020). Intergenerational transmission of diet-induced obesity, metabolic imbalance, and osteoarthritis in mice. Arthritis Rheumatol..

[7] Lohmander LS, Gerhardsson de Verdier M, Rollof J, Nilsson PM, Engstrom G (2009). Incidence of severe knee and hip osteoarthritis in relation to different measures of body mass: A population-based prospective cohort study. Ann. Rheum. Dis..

[8] Fain JN, Madan AK, Hiler ML, Cheema P, Bahouth SW (2004). Comparison of the release of adipokines by adipose tissue, adipose tissue matrix, and adipocytes from visceral and subcutaneous abdominal adipose tissues of obese humans. Endocrinology.

[9] Neumann E, Frommer KW, Muller-Ladner U (2014). Adiponectin as target in rheumatoid arthritis. Z Rheumatol..

[10] Hu E, Liang P, Spiegelman BM (1996). AdipoQ is a novel adipose-specific gene dysregulated in obesity. J. Biol. Chem..

[11] Trujillo ME, Scherer PE (2005). Adiponectin–journey from an adipocyte secretory protein to biomarker of the metabolic syndrome. J. Intern. Med..

[12] Ehling A (2006). The potential of adiponectin in driving arthritis. J. Immunol..

[13] Zuo W (2011). Adiponectin receptor 1 mediates the difference in adiponectin- induced prostaglandin E2 production in rheumatoid arthritis and osteoarthritis synovial fibroblasts. Chin. Med. J..

[14] Neumann E, Frommer KW, Vasile M, Muller-Ladner U (2011). Adipocytokines as driving forces in rheumatoid arthritis and related inflammatory diseases?. Arthritis Rheum..

[15] Yang WH (2013). Leptin induces IL-6 expression through OBRl receptor signaling pathway in human synovial fibroblasts. PLoS ONE.

[16] Tong KM (2008). Leptin induces IL-8 expression via leptin receptor, IRS-1, PI3K, Akt cascade and promotion of NF-kappaB/p300 binding in human synovial fibroblasts. Cell Signal.

[17] Jacobsen K (1977). Osteoarthrosis following insufficiency of the cruciate ligaments in man. A clinical study. Acta Orthop. Scand..

[18] Roos H, Adalberth T, Dahlberg L, Lohmander LS (1995). Osteoarthritis of the knee after injury to the anterior cruciate ligament or meniscus: The influence of time and age. Osteoarthr. Cartil..

[19] Glasson SS, Blanchet TJ, Morris EA (2007). The surgical destabilization of the medial meniscus (DMM) model of osteoarthritis in the 129/SvEv mouse. Osteoarthr. Cartil..

[20] Surwit RS, Kuhn CM, Cochrane C, McCubbin JA, Feinglos MN (1988). Diet-induced type II diabetes in C57BL/6J mice. Diabetes.

[21] West DB, Boozer CN, Moody DL, Atkinson RL (1992). Dietary obesity in nine inbred mouse strains. Am. J. Physiol..

[22] West KM, Kalbfleisch JM (1971). Influence of nutritional factors on prevalence of diabetes. Diabetes.

[23] Won, Y., Yang, J. I., Park, S. & Chun, J. S. Lipopolysaccharide binding protein and CD14, cofactors of toll-like receptors, are essential for low-grade inflammation-induced exacerbation of cartilage damage in mouse models of posttraumatic osteoarthritis. *Arthritis Rheumatol.* **73**(8), 1451–1460 (2021). 10.1002/art.41679 10.1002/art.41679 PMC8362181 33559324

[24] Mooney RA, Sampson ER, Lerea J, Rosier RN, Zuscik MJ (2011). High-fat diet accelerates progression of osteoarthritis after meniscal/ligamentous injury. Arthritis Res. Ther..

[25] Kimmerling KA (2020). Transgenic conversion of omega-6 to omega-3 polyunsaturated fatty acids via fat-1 reduces the severity of post-traumatic osteoarthritis. Arthritis Res. Ther..

[26] Wu CL (2015). Dietary fatty acid content regulates wound repair and the pathogenesis of osteoarthritis following joint injury. Ann. Rheum. Dis..

[27] Wu CL, Kimmerling KA, Little D, Guilak F (2017). Serum and synovial fluid lipidomic profiles predict obesity-associated osteoarthritis, synovitis, and wound repair. Sci. Rep..

[28] Pereira SS, Alvarez-Leite JI (2014). Low-grade inflammation, obesity, and diabetes. Curr. Obes. Rep..

[29] Xu H (2003). Chronic inflammation in fat plays a crucial role in the development of obesity-related insulin resistance. J. Clin. Investig..

[30] Warmink K (2020). High-fat feeding primes the mouse knee joint to develop osteoarthritis and pathologic infrapatellar fat pad changes after surgically induced injury. Osteoarthr. Cartil..

[31] Sun AR (2019). Pro-resolving lipid mediator ameliorates obesity induced osteoarthritis by regulating synovial macrophage polarisation. Sci. Rep..

[32] Abella V (2017). Leptin in the interplay of inflammation, metabolism and immune system disorders. Nat. Rev. Rheumatol..

[33] Pehlivanov B, Mitkov M (2009). Serum leptin levels correlate with clinical and biochemical indices of insulin resistance in women with polycystic ovary syndrome. Eur. J. Contracept. Reprod. Health Care.

[34] Considine RV (1996). Serum immunoreactive-leptin concentrations in normal-weight and obese humans. N. Engl. J. Med..

[35] Dall M (2018). Hepatic NAD(+) levels and NAMPT abundance are unaffected during prolonged high-fat diet consumption in C57BL/6JBomTac mice. Mol. Cell Endocrinol..

[36] Mousavi Z (2017). Correlation of visfatin level with non-alcoholic fatty liver in metabolic syndrome. Med. J. Islam Repub. Iran.

[37] Ku JH (2009). Correlation of synovial fluid leptin concentrations with the severity of osteoarthritis. Clin. Rheumatol..

[38] Junker S (2017). Expression of adipokines in osteoarthritis osteophytes and their effect on osteoblasts. Matrix Biol..

[39] Bas S (2014). Adipokines correlate with pain in lower limb osteoarthritis: Different associations in hip and knee. Int. Orthop..

[40] Beltowski J, Jamroz-Wisniewska A, Widomska S (2008). Adiponectin and its role in cardiovascular diseases. Cardiovasc. Hematol. Disord. Drug Targets.

[41] Chen HT (2014). Adiponectin enhances intercellular adhesion molecule-1 expression and promotes monocyte adhesion in human synovial fibroblasts. PLoS ONE.

[42] Wu MH, Tsai CH, Huang YL, Fong YC, Tang CH (2018). Visfatin promotes IL-6 and TNF-alpha production in human synovial fibroblasts by repressing miR-199a-5p through ERK, p38 and JNK signaling pathways. Int. J. Mol. Sci..

[43] Evans L, Williams AS, Hayes AJ, Jones SA, Nowell M (2011). Suppression of leukocyte infiltration and cartilage degradation by selective inhibition of pre-B cell colony-enhancing factor/visfatin/nicotinamide phosphoribosyltransferase: Apo866-mediated therapy in human fibroblasts and murine collagen-induced arthritis. Arthritis Rheum..

[44] Rosenbaum M, Leibel RL (1999). The role of leptin in human physiology. N. Engl. J. Med..

[45] Maeda N (2002). Diet-induced insulin resistance in mice lacking adiponectin/ACRP30. Nat. Med..

[46] Endisha H (2021). MicroRNA-34a-5p promotes joint destruction during osteoarthritis. Arthritis Rheumatol..

[47] Sun AR (2021). Effects of diet induced weight reduction on cartilage pathology and inflammatory mediators in the joint tissues. Front. Med..

[48] Duran S, Cavusoglu M, Kocadal O (2014). Ossification of the discoid meniscus: A case report. J. Clin. Orthop. Trauma.

[49] Rohilla S, Yadav RK, Singh R, Devgan A, Dhaulakhandi DB (2009). Meniscal ossicle. J. Orthop. Traumatol..

[50] Van Breuseghem I, Geusens E, Pans S, Brys P (2003). The meniscal ossicle revisited. Jbr-Btr.

[51] Yoo JH, Yang BK, Son BK (2007). Meniscal ossicle: A case report. Knee.

[52] Kapadia RD (2000). Meniscal ossification in spontaneous osteoarthritis in the guinea-pig. Osteoarthr. Cartil..

[53] Ernest TL, Kondrashov PE (2018). The role of excessive body weight and meniscal instability in the progression of osteoarthritis in a rat model. Knee.

[54] Neumann L (2008). A cross-sectional study of the relationship between body mass index and clinical characteristics, tenderness measures, quality of life, and physical functioning in fibromyalgia patients. Clin. Rheumatol..

[55] Glasson SS, Chambers MG, Van Den Berg WB, Little CB (2010). The OARSI histopathology initiative—Recommendations for histological assessments of osteoarthritis in the mouse. Osteoarthr. Cartil..

[56] Gilat T (2003). Prevention of diet-induced fatty liver in experimental animals by the oral administration of a fatty acid bile acid conjugate (FABAC). Hepatology.

[57] Brunt EM (2009). Portal chronic inflammation in nonalcoholic fatty liver disease (NAFLD): A histologic marker of advanced NAFLD-Clinicopathologic correlations from the nonalcoholic steatohepatitis clinical research network. Hepatology.

[58] Kleiner DE (2005). Design and validation of a histological scoring system for nonalcoholic fatty liver disease. Hepatology.

