# Supplementary material for: Systemic versus local adipokine expression differs in a combined obesity and osteoarthritis mouse model
Source: Sci Rep. 2021 Aug 20;11:17001. doi: 10.1038/s41598-021-96545-8 (PMC8379250; doi:10.1038/s41598-021-96545-8)
Supplement: Supplementary file 1 — Supplementary Information 1. [file 41598_2021_96545_MOESM1_ESM.pdf]

Title: Systemic versus local adipokine expression differs in a combined obesity and osteoarthritis mouse model

Authors: Marie-Lisa Hülser, Yubin Luo, Klaus Frommer, Rebecca Hasseli, Kernt Köhler, Magnus Diller, Lina Van Nie, Christoph Rummel, Martin Roderfeld, Elke Roeb, Georg Schett, Aline Bozec, Ulf Müller-Ladner, Elena Neumann

Supplement 1:

a 4 weeks 6 weeks 8 weeks

ND

HFD

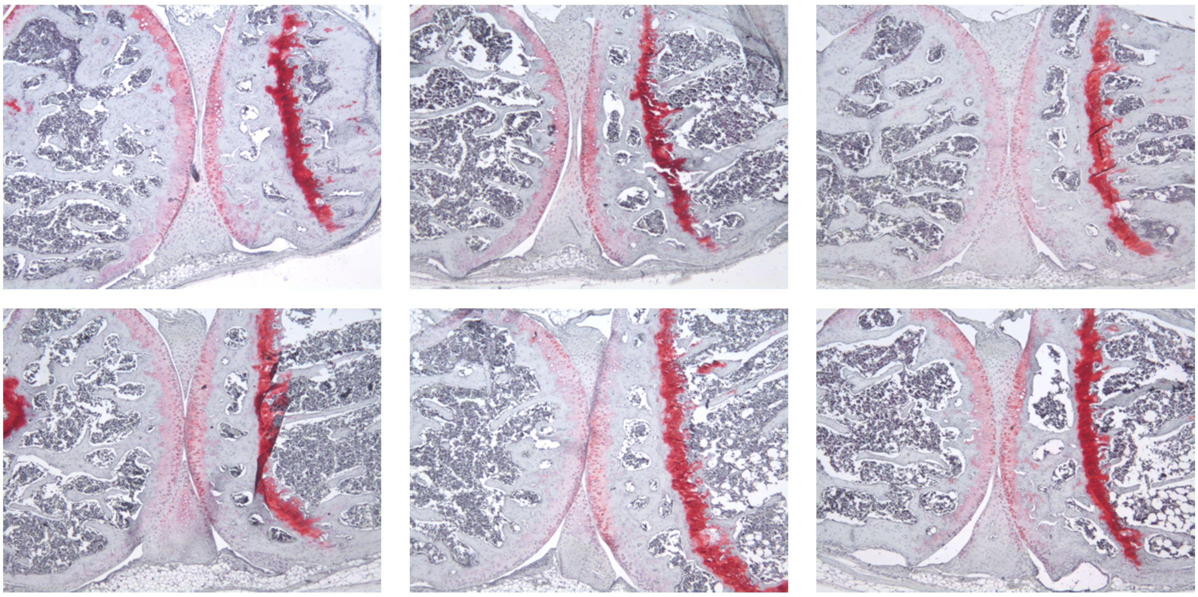

b 4 weeks 6 weeks 8 weeks

ND

HFD

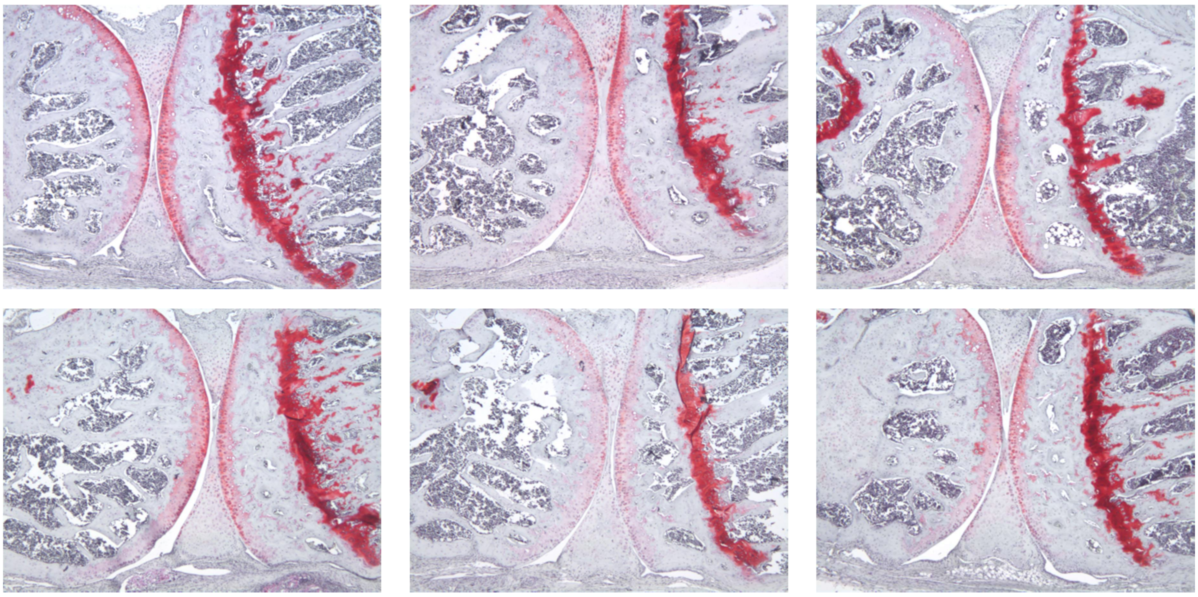

c

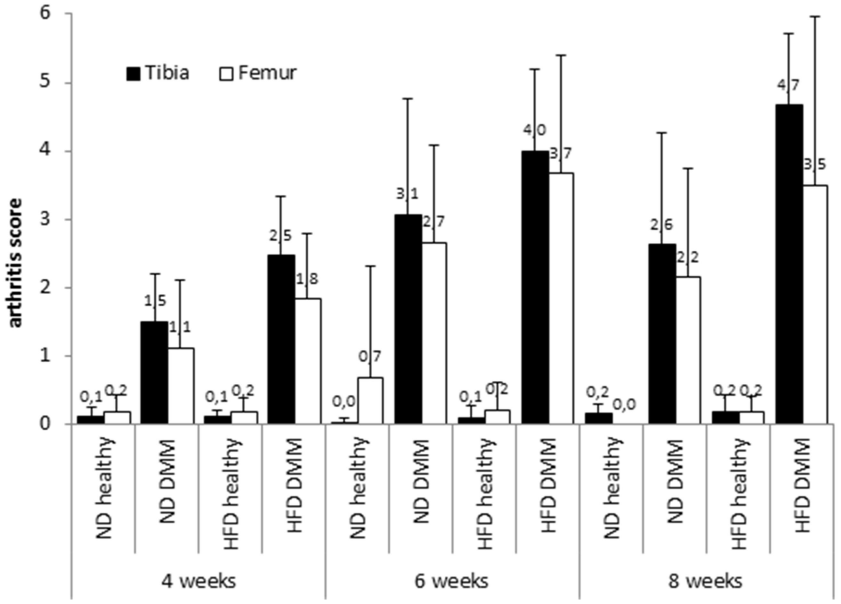

Safranin-O staining of healthy untreated control animals (a) and of sham-treated surgery control limbs of the DMM animals (b) for ND and HFD after 4, 6 and 8 weeks, respectively. Representative images in 50-fold magnification, left: femur, right: tibia. **c) Arthritis score of the tibia (black bars) compared to the femur (white bars) showing comparable scores.**
