# Supplementary material for: Systemic versus local adipokine expression differs in a combined obesity and osteoarthritis mouse model
Source: Sci Rep. 2021 Aug 20;11:17001. doi: 10.1038/s41598-021-96545-8 (PMC8379250; doi:10.1038/s41598-021-96545-8)
Supplement: Supplementary file 2 — Supplementary Information 2. [file 41598_2021_96545_MOESM2_ESM.pdf]

Title: Systemic versus local adipokine expression differs in a combined obesity and osteoarthritis mouse model

Authors: Marie-Lisa Hülser, Yubin Luo, Klaus Frommer, Rebecca Hasseli, Kernt Köhler, Magnus Diller, Lina Van Nie, Christoph Rummel, Martin Roderfeld, Elke Roeb, Georg Schett, Aline Bozec, Ulf Müller-Ladner, Elena Neumann

Supplement 2:

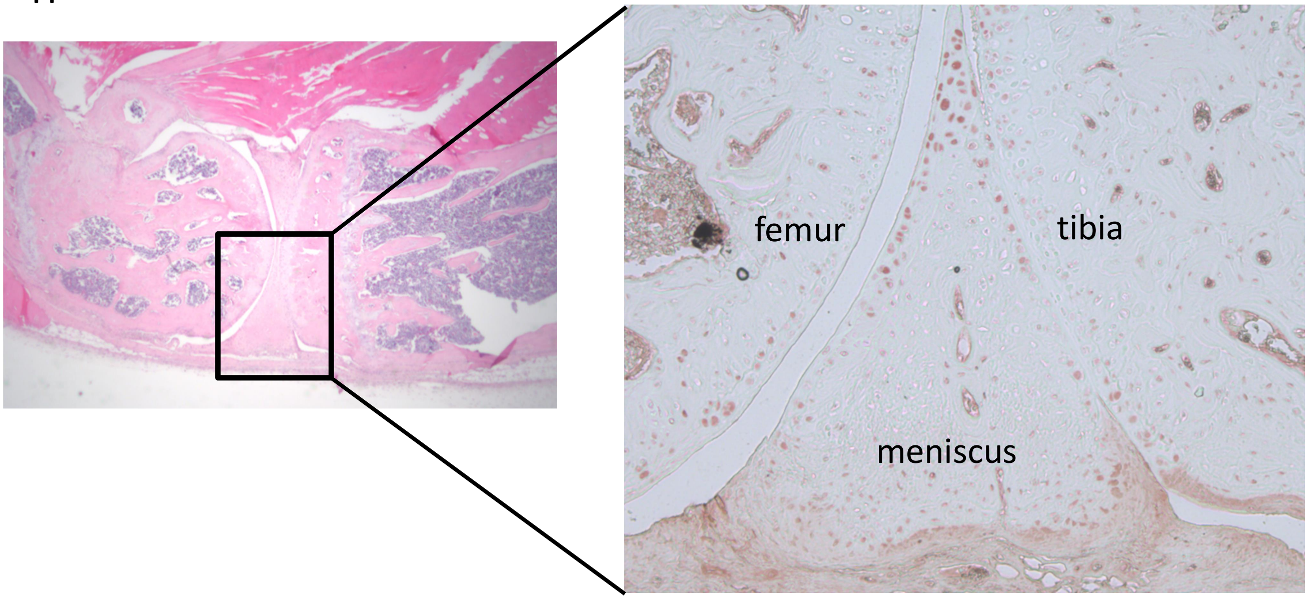

The quantification of leptin- or adiponectin-positive cells was performed in the ventral meniscus as indicated above. All cells located in the meniscus were quantified as well as adiponectin-positive cells stained by immunohistochemistry. The proportion of adiponectin-positive cells vs. the total cell number was calculated (in %). For leptin, cells strongly expressing leptin were quantified as a low leptin expression was visible for most cells vs. the total cell number (in %).
