# Supplementary material for: Systemic versus local adipokine expression differs in a combined obesity and osteoarthritis mouse model
Source: Sci Rep. 2021 Aug 20;11:17001. doi: 10.1038/s41598-021-96545-8 (PMC8379250; doi:10.1038/s41598-021-96545-8)
Supplement: Supplementary file 3 — Supplementary Information 3. [file 41598_2021_96545_MOESM3_ESM.pdf]

Title: Systemic versus local adipokine expression differs in a combined obesity and osteoarthritis mouse model

Authors: Marie-Lisa Hülser, Yubin Luo, Klaus Frommer, Rebecca Hasseli, Kernt Köhler, Magnus Diller, Lina Van Nie, Christoph Rummel, Martin Roderfeld, Elke Roeb, Georg Schett, Aline Bozec, Ulf Müller-Ladner, Elena Neumann

Supplement 3:

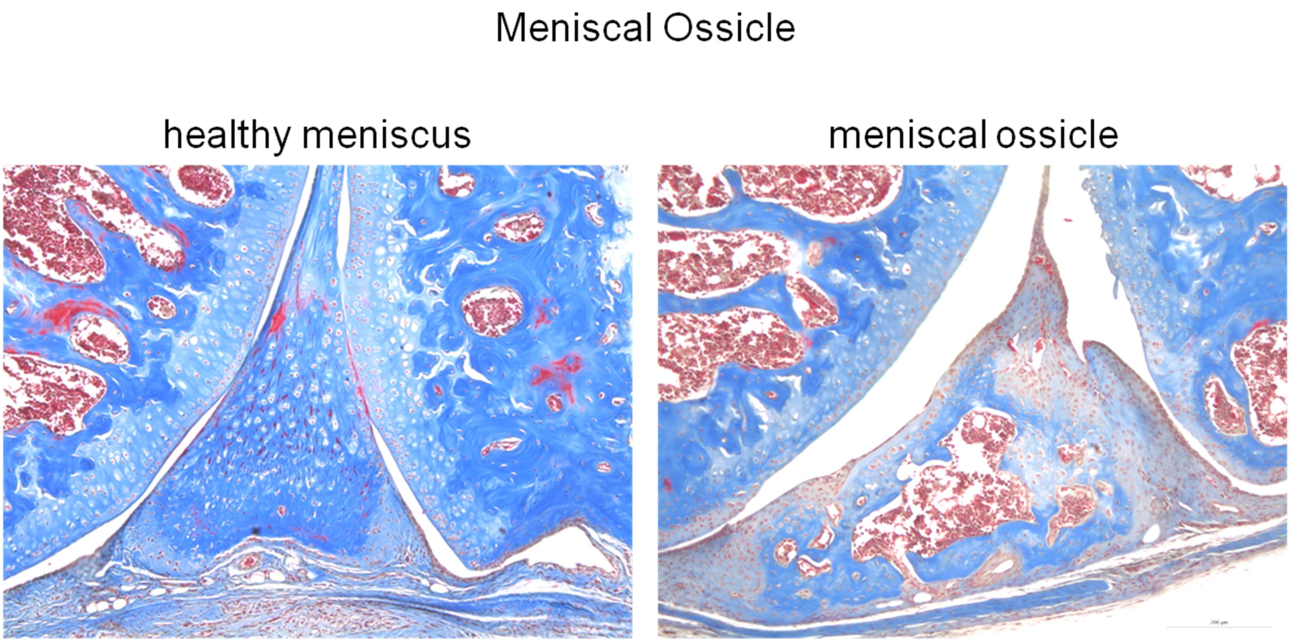

Masson-Goldner staining of the meniscus with (right) and without (left) meniscal ossicle. Representative images. (100-fold magnification)
