# Supplementary material for: Systemic versus local adipokine expression differs in a combined obesity and osteoarthritis mouse model
Source: Sci Rep. 2021 Aug 20;11:17001. doi: 10.1038/s41598-021-96545-8 (PMC8379250; doi:10.1038/s41598-021-96545-8)
Supplement: Supplementary file 4 — Supplementary Information 4. [file 41598_2021_96545_MOESM4_ESM.pdf]

Title: Systemic versus local adipokine expression differs in a combined obesity and osteoarthritis mouse model

Authors: Marie-Lisa Hülser, Yubin Luo, Klaus Frommer, Rebecca Hasseli, Kernt Köhler, Magnus Diller, Lina Van Nie, Christoph Rummel, Martin Roderfeld, Elke Roeb, Georg Schett, Aline Bozec, Ulf Müller-Ladner, Elena Neumann

Supplement 4:

Supplement 4, Table 1: Two-way ANOVA results for the effects of diet (ND/HFD) and DMM-mediated OA induction on OA score, liver score and NASH CRN score (cf. Figure 1d/e/f)

**Parameter**	**Time point**	**P values and significance levels**			
		Factor		Interaction (diet X OA induction)	Significant interaction?
		Diet	OA induction		
OA score (right leg tibia)	4 weeks	0.0488 (*)	<0.0001 (***)	0.0496 (*)	yes
	6 weeks	0.2354	<0.0001 (***)	0.3165	no
	8 weeks	0.0132 (*)	<0.0001 (***)	0.0150 (*)	yes
Liver score	4 weeks	<0.0001 (***)	0.0589	0.0042 (**)	yes
	6 weeks	<0.0001 (***)	0.3823	0.0285 (*)	yes
	8 weeks	<0.0001 (***)	0.0249 (*)	0.3049	no
NASH CRN score	4 weeks	0.0082 (**)	0.0002 (***)	0.0082 (**)	yes
	6 weeks	<0.0001 (***)	0.0022 (**)	0.0022 (**)	yes
	8 weeks	0.0001 (***)	0.1436	0.1436	no

Supplement 4, Table 2: Two-way ANOVA results for the effects of diet (ND/HFD) and DMM-mediated OA induction on adipokine serum levels (cf. Figure 2)

**Parameter**	**Time point**	**P values and significance levels**			
		Factor		Interaction (diet X OA induction)	Significant interaction?
		Diet	OA induction		
adiponectin serum level	4 weeks	0.8402	0.1651	0.5950	no
	6 weeks	0.0538	0.0611	0.9375	no
	8 weeks	0.0074 (**)	0.0006 (***)	0.0181 (*)	yes
leptin serum level	4 weeks	<0.0001 (***)	<0.0001 (***)	0.0066 (**)	yes
	6 weeks	<0.0001 (***)	0.0347 (*)	0.1558	no
	8 weeks	0.0007 (***)	0.0786	0.0966	no
visfatin serum level	4 weeks	0.1696	0.4393	0.7011	no
	6 weeks	0.3377	0.6684	0.9151	no
	8 weeks	0.0962	0.5759	0.1417	no

ND: normal diet; HFD: high-fat diet; DMM: destabilization of the medial meniscus; OA: osteoarthritis; NASH: non-alcoholic steatohepatitis; CRN: Clinical Research Network / Significance: p<0.05= *; p<0.01=**; p<0.001=***
